# Unity Is Intelligence: A Collective Intelligence Experiment on ECG Reading to Improve Diagnostic Performance in Cardiology

**DOI:** 10.3390/jintelligence9020017

**Published:** 2021-04-01

**Authors:** Luca Ronzio, Andrea Campagner, Federico Cabitza, Gian Franco Gensini

**Affiliations:** 1 Dipartimento di Informatica, Sistemistica e Comunicazione, University of Milano-Bicocca, Viale Sarca 336, 20126 Milan, Italy; l.ronzio@campus.unimib.it (L.R.); a.campagner@campus.unimib.it (A.C.); 2 IRCCS MultiMedica, Sesto San Giovanni, 20099 Milan, Italy; gfgensini@gmail.com

**Keywords:** collective intelligence, ECG reading, medical decision support, diagnostic error

## Abstract

Medical errors have a huge impact on clinical practice in terms of economic and human costs. As a result, technology-based solutions, such as those grounded in artificial intelligence (AI) or collective intelligence (CI), have attracted increasing interest as a means of reducing error rates and their impacts. Previous studies have shown that a combination of individual opinions based on rules, weighting mechanisms, or other CI solutions could improve diagnostic accuracy with respect to individual doctors. We conducted a study to investigate the potential of this approach in cardiology and, more precisely, in electrocardiogram (ECG) reading. To achieve this aim, we designed and conducted an experiment involving medical students, recent graduates, and residents, who were asked to annotate a collection of 10 ECGs of various complexity and difficulty. For each ECG, we considered groups of increasing size (from three to 30 members) and applied three different CI protocols. In all cases, the results showed a statistically significant improvement (ranging from 9% to 88%) in terms of diagnostic accuracy when compared to the performance of individual readers; this difference held for not only large groups, but also smaller ones. In light of these results, we conclude that CI approaches can support the tasks mentioned above, and possibly other similar ones as well. We discuss the implications of applying CI solutions to clinical settings, such as cases of augmented ‘second opinions’ and decision-making.

## 1. Introduction

Diagnostic and therapeutic errors are far from rare; it is estimated that 10–15% of all reported diagnoses are incorrect Graber (2013), and this entails millions of diagnostic errors each year in healthcare systems throughout the world Newman-Toker et al. (2020). The World Health Organization (WHO) estimates that the cost of medical errors represents almost one quarter of a country’s health expenditures World Health Organization et al. (2018). Even in a consolidated medical exam such as electrocardiography (ECG) and for a relatively common condition like atrial fibrillation, a large review of ECGs in a community hospital found that almost 8% of cases were misdiagnosed Davidenko and Snyder (2007).

However, medical errors are not necessarily a manifestation of malpractice or negligence Gruver and Freis (1957), as they mostly depend on the complexity of the context in which health workers have to operate every day, which places them under conditions of great strain and uncertainty. For this reason, there has always been great hope in the role of technology in reducing variability, helping to cope with uncertainty, and creating new working conditions and forms of collaboration among health practitioners. To achieve these broad aims, IT-based solutions Topol (2019) have been proposed to mitigate error rates in clinical practice. These solutions can be implemented either by using artificial intelligence (AI) tools to support cognitive tasks and decision-making Quer et al. (2017), or by leveraging collaborative tools to support knowledge sharing or its combination, as in the case of collective intelligence (CI) solutions Tucker et al. (2019).

In this paper, we focus on the latter kind of technology-mediated support. We interpret CI as the ‘collective insight of groups working on a task [that] has the potential to generate more accurate information or decisions than individuals can make alone’ Radcliffe et al. (2019). This phenomenon has long been studied by many scholars (e.g., Hong and Page 2012; Surowiecki 2004. Specifically, this approach to knowledge elicitation and collective resolution has also been recently studied in the medical field as a means of improving clinicians’ diagnostic accuracy (some of the main studies in this area include Wolf et al. (2015); Kurvers et al. (2015); Kattan et al. (2016); Kammer et al. (2017); Barnett et al. (2019); Hernández-Chan et al. (2012, 2016)).

Multiple approaches have been devised to exploit the intelligence emerging from a collective of clinical raters gathered to express their views of a particular case Gregg (2010), ranging from those characterized by continuous feedback among the raters and their synchronous and co-located interaction and mutual influence Bahrami et al. (2010); Rosenberg et al. (2018), to approaches based on the aggregation of the opinions of different experts who do not directly interact with each other Barnett et al. (2019). Medical practice is replete with examples of the first kind, which include clinical case discussions Launer (2016) on everyday occasions when doctors get together in small teams to evaluate, collect advice, and reach decisions on medical case histories. In this paper, we focus on the latter approach, which we denote with the generic expression of CI. We focus on CI for its potential to make the ‘meeting of brains’ mentioned above more efficient and structured than in synchronous meetings. These meetings, although likely the richest form of group interaction and collaboration, can be an unpractical form of organization to address each informative need or clinical question when they arise, or they can be excessively time consuming, given the frantic schedules, busy workloads and unexpected emergencies that characterize hospital settings. They can also be simply impossible to organize within the less collaborative nature of primary settings, such as clinics and counselling centres.

Furthermore, synchronous, co-located, and interactive protocols could pose the grand challenges that are discussed in the literature on the psychology of groups Dinh and Salas (2017) and consensus issues, such as the Delphi methods Steurer (2011), and would thus be subject to group effects and biases such as groupthink Kaba et al. (2016), the bandwagon effect O’Connor and Clark (2019), and authority bias Howard (2019).

While ’intermediate’, asynchronous, and interactive CI approaches, such as peer reviewing or contested CI De Liddo et al. (2012), may be similarly exempt from these limitations, we focus in this article on fully non-interactive CI protocols, since these approaches are typically faster to set up and converge upon an actionable group decision. This latter characteristic makes these protocols more suitable for emergency settings, especially when the case at hand can be properly represented in a concise and simple way (as case histories usually are), and the issues upon which a consensus must be reached are just as simple (e.g., choosing an investigation, diagnosis, or treatment option). This is because they can be traced back to the choice of an option among a limited number of possible alternatives.

At the same time, to be applied to diagnostic tasks, such CI protocols require digital platforms that could invite raters to join the collective effort, present the clinical cases on demand, collect opinions from multiple raters in some codified manner (e.g., by allowing raters to choose among a set of options that are possibly predefined by a referring clinician), and then process these ratings so as to identify the emerging ‘best’ (or simply more representative) option from this collective Hernández-Chan et al. (2012, 2016); Maglogiannis et al. (2006).

In what follows, we report on a CI experiment in which multiple readers (including medical school students and young clinicians) were invited to read a number of ECGs and interpret the corresponding cardiological cases by means of a simple online questionnaire. The goal of this experiment was to assess whether CI could improve diagnostic accuracy and reduce diagnostic errors in a realistic (although simulated) business use case. To our knowledge, this is the first experiment of this kind that applies CI principles to ECG reading and interpretation.

## 2. Methodology

### 2.1. Data Collection

Each participant in the study was tasked with the interpretation of a collection of ECGs by means of an online questionnaire that was developed on the Limesurvey platform (version 3.23.1). The ECGs were selected among those available in the Wave-Maven (WM) educational database Nathanson et al. (2001). Each ECG in the WM repository is associated with a brief description of the findings in the electrocardiographic trace and a difficulty rating (from 1 to 5), which we mapped onto a simpler three-point scale (i.e., easy, medium, and difficult). From this comprehensive dataset of more than 500 cases, we selected 10 ECGs sorted by difficulty in the following way: two were easy, six were medium, and two were difficult. Information about the selected ECGs is reported in Table 1.

We randomly assigned each ECG to one of two groups, with each encompassing five ECGs; each participant in the study was asked to interpret the cases from one of these two groups of ECGs.

The questionnaire was made available to the participants through the Limesurvey LimeSurvey Project Team/Carsten Schmitz (2012) platform for 14 days, from 17 to 30 January 2020. During this time, the survey was advertised among the students of the Faculty of Medicine and Surgery at the University of Milano-Bicocca, recent graduates and interns of the same university, and a convenience sample of students and doctors. The number of readers for each ECG and their level of reported experience is reported in Table 2.

As indicated above, each participant was supposed to consider five ECGs and case summaries: for each ECG, the participant was invited to write the diagnosis or main finding in a free-text field. The participants were also asked to report their expertise level; their confidence in the answers (according to an ordinal scale ranging from 1 at the lowest to 6 at the highest level of confidence); whether they had followed intuition or applied the standardized criteria from the guidelines; and, lastly, the perceived level of difficulty of each case.

After collection, the free-text responses of the participants were converted into a structured dataset by one of the authors by referring to the established guidelines taken from manuals and textbooks Hancock et al. (2009); Macfarlane et al. (2010); Rautaharju et al. (2009); Surawicz et al. (2009); Wagner et al. (2009). This author coded each answer in terms of two possible responses: 1 for the presence of a given finding, or 0 for its absence. The same coding scheme was also applied to the ground-truth diagnosis and the findings reported in the WM ECG repository; the participants’ structured responses were then compared with the correct responses in order to determine their accuracy and completeness.

### 2.2. Collective Intelligence Protocols

The goal of our study was to assess whether a CI approach could improve the diagnostic accuracy of a group of ECG readers. To achieve this aim, we implemented a computational procedure to aggregate the responses of the single participants of the study. We considered virtual CI teams of differing group sizes, ranging from just three annotators (the minimal amount to get a majority vote) to as many as 30. This allowed us to assess whether there was any correlation (if any) between group size and accuracy improvement with respect to the single decisions. Moreover, we decided to evaluate the following three different aggregation protocols, which had been applied to the collected responses:

- Frequency: The responses of the group members were ranked according to their frequency among the members of the group.
- Experience: The responses of each member of the group were weighted proportionally to their experience and then ranked according to their aggregated weight.
- Confidence: The responses of each member of the group were weighted proportionally to the confidence they expressed and then ranked according to their aggregated weight.

In all the three cases mentioned above, ties among the different responses were resolved by randomly selecting just one of the tied options.

In order to evaluate the diagnostic accuracy of both the single participants and the aggregated groups, we considered two different metrics: strict accuracy (SA) and loose accuracy (LA). For any group and ECG, SA was defined by comparing the mode of the responses (i.e., the most frequently chosen one) of the group (according to one of the three aggregation protocols described above) with the reference diagnosis (taken as a correct gold standard). LA was defined by taking into account the five most frequently chosen responses for each group; the group response was deemed *loosely correct* if the gold-standard diagnosis was mentioned among these five options.

**Figure 1:**
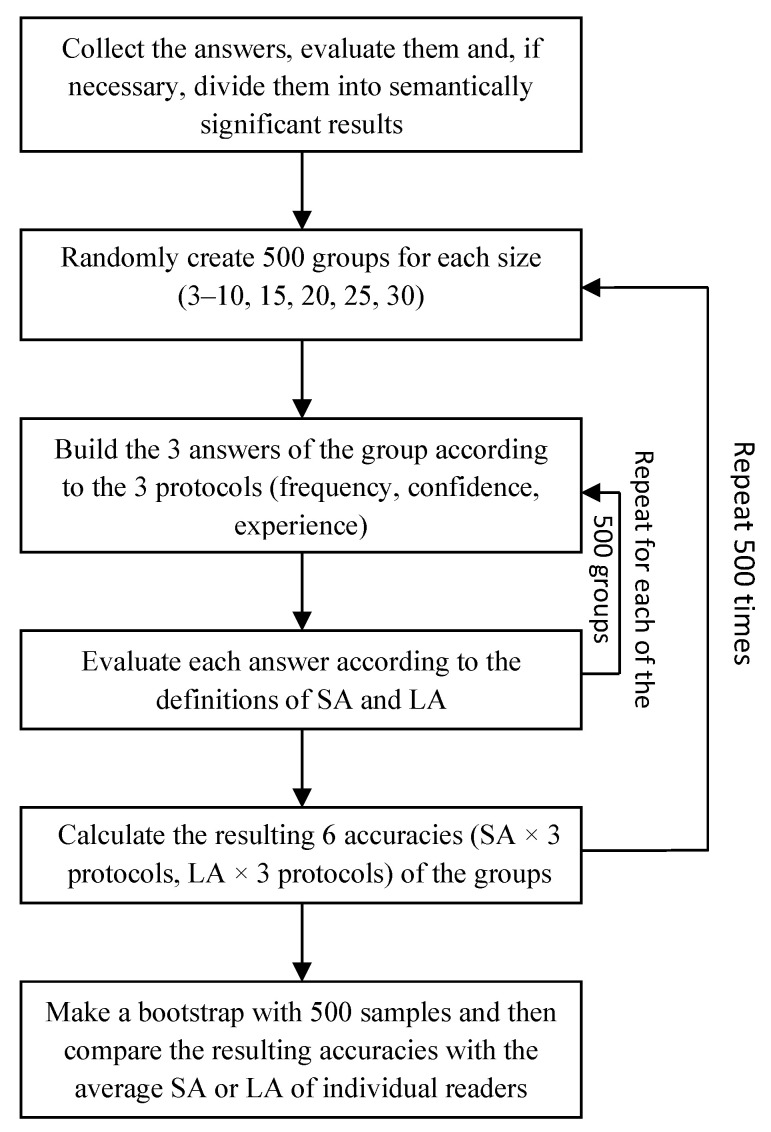
Flow-chart diagram of the bootstrap procedure implemented to compute the accuracy of the CI groups.

Since computing the exact average accuracy (both LA and SA) for all CI groups would have been computationally infeasible, we approximated these scores through an estimation approach based on the non-parametric bootstrap procedure Efron (1979). This procedure is reported in Figure 1.

For a total number of $N_{cycles}=500$ iterations, we performed the following operations:

- We sampled $N_{samples}=500$ groups of size $N_{groups}$ (ranging from three to 30) by extracting (uniformly at random) $N_{groups}$ readers from the set of all respondants;
- For each case and each sampled group, we computed the group answers according to the three considered protocols;
- We evaluated the accuracy (both LA and SA) of each of the $N_{samples}$ sampled groups according to all three protocols;
- We computed the average group accuracy (both LA and SA for each aggregation protocol) by averaging across the $N_{samples}$ sampled groups.

As a consequence of this procedure, we obtained a collection of 500 average accuracy scores for each aggregation protocol and accuracy metric, which resulted in a total of 3000 average accuracy scores. The $N_{cycles}=500$ accuracy estimates were re-sampled with replacements, which generated $N_{boots}=500$ resamples. The bootstrap estimates of average accuracy were computed for these resamples, along with the standard deviation, median, and 95% confidence intervals. We refer those interested in more details about this estimation procedure and its statistical properties to Efron (1979); Efron and Tibshirani (1994).

For the individual readers, their response on a given ECG was considered strictly accurate (cf. SA) if the respondent had given only the correct answer, and loosely accurate (cf. LA) if this correct answer was selected among other possibly incorrect findings. The average SA and LA scores of the individuals and groups were compared by means of a *t*-test; findings associated with a two-tailed *p*-value lower than 0.05 were considered significant. The statistical analyses were performed using R (version 3.7.0).

## 3. Results

**Figure 2:**
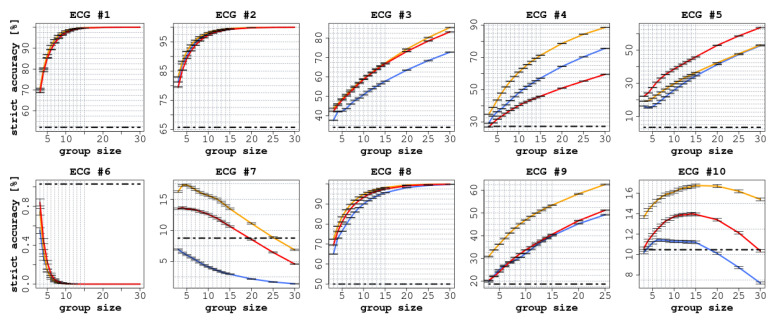
Strict accuracy (SA) of the 10 ECGs with increasing group size. In each diagram, the red line represents the experience aggregation protocol, the yellow line represents the confidence aggregation protocol, and the blue line represents the frequency aggregation protocol. The black-dotted line represents the average performance of the individual readers.

**Figure 3:**
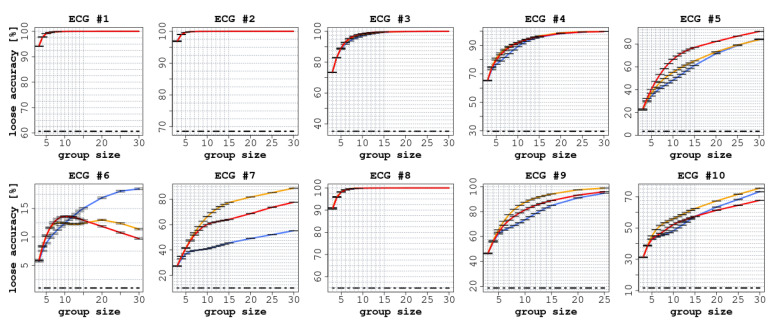
Loose accuracy (LA) of the 10 ECGs with increasing group size. In each graph, the red line represents the experience aggregation protocol, the yellow line represents the confidence aggregation protocol, and the blue line represents the frequency aggregation protocol. The black dotted line represents the average performance of the individual readers.

Each ECG had a different number of readers (between 75 and 140), as shown in Table 2. In all ECGs, we observed a significant improvement in accuracy (both SA and LA) when comparing the performance of individual readers with the CI groups, as reported in Figure 2 and Figure 3.

In terms of SA, we observed an improvement in all cases except for ECGs 6, 7, and 10 (in the latter case, only the confidence aggregation rules constantly improved when the group size increased). The improvements ranged from 30.8 percentage points (CI: 95% 30.3 ÷ 31.2) for ECG 9 to 61.3 (CI: 95% 60.9 ÷ 61.8) for ECG 4, as shown in Figure 2, and were statistically significant (p<0.0001).

Similarly, in terms of LA, we report an improvement in all the ECGs for all three of the considered CI protocols. As reported in Figure 3, the improvements ranged from 8.6 percentage points (confidence interval [CI]: 95% 8.5 ÷ 8.8) for ECG 6 to 87.5 (CI: 95% 87.3 ÷ 87.7) for ECG 5. In all cases, the improvement, with respect to the accuracy of the single participants, was statistically significant (p<0.0001).

The aggregation protocol providing the greatest improvement was the confidence protocol, as eight (4 SA and 4 LA) out of 20 results were statistically significantly better than the other aggregation protocols. The increase, excluding the cases reaching almost 100% for all the rules, ranged from 4.1 percentage points (CI: 95% 4.0 ÷ 4.2) for ECG 9 (confidence vs frequency) to 33.6 (CI: 95% 33.4 ÷ 33.8) for ECG 7 (confidence vs. frequency).

In contrast, experience only resulted in an improvement for two cases (1 SA and 1 LA) out of 20, with an increase of up to 11.0 (CI: 95% 10.7 ÷ 11.2) percentage points for ECG 5 (experience vs. frequency) with respect to the other rules, and a net decrease in three (1 SA and 2 LA) cases out of 20. In all cases, the improvement or the worsening in accuracy was statistically significant (p<0.0001).

## 4. Discussion

In recent years, the application of CI methods in medicine has attracted a significant amount of interest as a way to improve diagnostic accuracy and reduce diagnostic or therapeutic errors. For instance, Barnett et al. (2019) used data from the Human Diagnosis Project to show that CI methods can greatly reduce diagnostic errors. In another study, Fontil et al. (2019) proposed a CI platform to improve diagnoses in primary and urgent care settings, and Rinner et al. (2020) discussed a CI approach for discriminating between benign and malignant pigmented skin lesions. We refer the interested reader to the recent review by Radcliffe et al. (2019) for further examples and considerations.

In this article, we have discussed an application of CI protocols to ECG reading. In doing so, we aimed to address an important gap in the existing literature, since electrocardiography is an important and widely used examination in many different settings, including cardiology, emergency medicine, and routine patient health assessments Fye (1994); Sattar and Chhabra (2020). In fact, to the best of the authors’ knowledge, this is the first study applying a CI approach to ECG reading for open-ended diagnostic and annotation purposes. Indeed, while some previous works, such as the research by Zhu et al. (2014), have applied CI methods to ECGs, we note that the scope of our work is quite different and more general. In Zhu et al. (2014), the authors focus on using CI to annotate a very specific morphological pattern (QT interval), while in our work, CI methods are used to discover the correct findings and diagnosis, which are supplied as free-text annotations. Additionally, compared to Zhu et al. (2014), we considered a much larger and more diverse population of annotators.

Further, in contrast to other research Kammer et al. (2017); Kattan et al. (2016), this study did not have the participants select the presence/absence of a pathological condition from a list of options; rather, we asked the participants to report their findings in a more naturalistic way by writing a short report, as they would in a real-world diagnostic setting. The former scenario is plausible only in second-opinion settings where a referring clinician turns to a ‘crowd’ of colleagues after pre-selecting a number of potential diagnostic candidates, although this could omit the right answer and induce some priming effects unless the options are presented in random order. The latter scenario, which we chose, is more representative of two main cases: one in which a referring doctor proposes a case, but refrains from conditioning the colleagues’ interpretation by giving them the case without comments, and a second, more common case, where no referring doctor exists, and a team of doctors must collaborate to propose one (or few) diagnoses, which are then given to the responsible cardiologist on duty for the official report. In this latter class of scenarios, extracting the clinical findings from the participants’ reports poses a technological challenge related to natural language processing Assale et al. (2019), which we do not address here. It should be noted that not all business cases require such an extraction. In other words, we can see the output of a CI initiative, if systematized and considered part and parcel of the clinical workflow, as a multifaceted recommendation that can empower single accountable clinicians with superhuman abilities, with the insight being given by a ‘social machine’ that shares much more of the clinical context and specialist knowledge than any technological, ‘artificial intelligence’ entity ever could.

**Figure 4:**
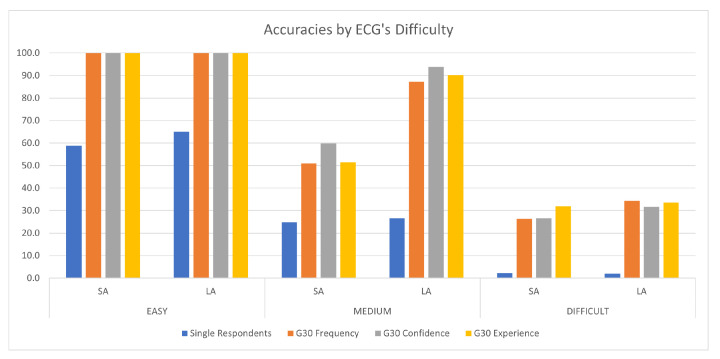
Average strict accuracy and loose accuracy for all the ECG difficulty levels. In the figure, we report the average accuracy for single respondents and groups of 30 (G30).

Thus, in our study we found that the application of CI can significantly improve the accuracy in the reading of ECGs with respect to individual performance; moreover, this improvement occurred for both simple and complex cases. Although it is intuitive that the improvement may occur in difficult ECGs, given the intrinsic challenges that these pose to human interpretation, the improvement in the results in easy ECGs was still relevant at 34∼50 percentage points (see Figure 4).

In addition, the improvement in accuracy was statistically, but also qualitatively, significant already for groups involving only three to five readers. We also observed that the improvement increased as the group size increased without necessarily reaching a plateau at 9 or 10 members, as observed in other studies Kattan et al. (2016). Consequently, it would be possible to significantly improve the diagnostic performance even by involving a relatively small number of individual clinicians, although larger improvements can be expected if more participants are involved. Furthermore, as we discuss in the following, we observed that even groups comprising only non-expert readers could provide improvements over the performance of single readers and even (single) experts.

Going into more detail on the reported results, we can see that the two evaluation metrics considered (SA and LA) entail two different perspectives on the goals of CI in diagnostic settings. On the one hand, CI can be used as a tool for ‘decision making’, since applying the protocol allows for the most plausibly correct answer to be selected. On the other hand, CI can be seen as a tool for ’decision support’ in the case of LA, as the protocols provide a set of plausible alternatives from which competent users can choose the most plausible option.

Focusing on SA (see Figure 2), we can identify three different situations. In ECGs 1, 2, and 8, which were cases of low-to-medium difficulty, the accuracy of the readers quickly reached a plateau around 100% for group sizes greater than 10, and a significant improvement was reached already with groups of three readers. This should not be surprising, as most of the individual readers correctly identified the right responses.

Conversely, in ECGs 3, 4, 9, and 5, the (strict) accuracy of the readers continued to grow as the size of the groups increased. In these cases, the greatest improvements were obtained, ranging from 44 (CI: 95% 43.7 ÷ 44.5) percentage points in ECG 9 to 61 (CI: 95% 60.9 ÷ 61.8) in ECG 4. ECG 5 in particular, which was considered ‘difficult’, is an interesting case to consider in appraising the potential of CI protocols, as only 3.5% of the individual readers provided a correct interpretation. This is likely because it was a complex case involving multiple findings; nonetheless, by aggregating the individual responses the accuracy increased almost linearly with the group size. This is a clear example of the potential that CI could reach in clinical settings when adopted as a decision-making tool.

At the same time, ECGs 6, 7, and 10 illustrate the risk of using CI as a decision-making tool. In these cases, the groups achieved worse results than individual readers, as for ECGs 7 and 10 the correct answer was given by only 10% of the individual readers. Hence, the correct response was progressively overcome in frequency by other (incorrect but more popular) responses. This implies that the more frequent, but incorrect, responses ended up being more common than the correct (but relatively infrequent) response. ECG 6, by contrast, was a complex multi-faceted case characterized by multiple possible findings. While some respondents identified certain aspects of the correct answer, almost no one was able to identify the main finding. We conjecture two possible explanations for this phenomenon. The first regards the fact that these ECGs were not characterised by multiple findings or a composite diagnosis; in fact, they were cases for which only a single correct finding existed. Therefore, the simple integration of multiple responses was not able to bring in any improvement with respect to individual answers, given that the correct finding was identified by only a small portion of the respondents. The second explanation regards the fact that these ECGs were of the kind known as ’seen one, seen them all’, and were probably not in the shared background of most readers (who, we can recall, were mostly trained at the same institution). To this end, the application of semi-interactive CI protocols, such as intermittent interactive CI Bernstein et al. (2018) or the ’surprisingly popular’ approach Prelec et al. (2017), may help in identifying the knowledgeable raters and thus improve the performance of the group. Indeed, these methods have recently been proven to be effective in medical settings as well Gitto et al. (2020). While we focused in this article on standard, better-understood, and fully non-interactive protocols, we believe that further research should aim at exploring these alternative protocols.

Alternative protocols notwithstanding, our results highlight the major limitation of CI as a decision-making tool, which is the risk of incurring in a lack of independence among the readers. In these cases, no matter the group size or the adopted aggregation protocol, if the predominant idea in a population is wrong (as in some cases above), then SA will always tend toward 0% accuracy Koriat (2012, 2015). This is the main reason why we also considered LA. Specifically, we proposed LA as a novel metric that shows how CI protocols can be used to support diagnostic tasks in a ’crowd-sourced’ *second opinion* service: the clinician, when in doubt about the correct interpretation of any new ECG, turns to the ’crowd’, and from the participants’ answers a list of candidate answers (e.g., five in our experiments) is extracted and given to the clinician as plausible alternatives. This CI protocol is similar to what is performed by crowd-sourcing platforms such as CrowdMed (https://www.crowdmed.com/, accessed on 23 March 2021) Meyer et al. (2016). In this study, we provide an empirical demonstration of the feasibility and potential effectiveness of these solutions.

With regard to LA, we observed a clear improvement in all cases (see Figure 3). In six ECGs out of 10, the CI protocol reached an LA value close to 100%; the improvement, with respect to the individual readers, was always significant. For instance, in ECG 5, LA reached 88% (CI: 95% 87.3 ÷ 87.7 p<0.0001), and in ECG 9, it reached 81% (CI: 95% 80.1 ÷ 80.8 p<0.0001). In the three ECGs where we did not detect an improvement in terms of SA, such an improvement was nevertheless detected in terms of LA. In ECG 6, LA grew by 17 percentage points (CI: 95% 17.2 ÷ 17.5), although the correct answers were indicated only by a minority of readers, whereas in ECG 7 LA increased by 79 percentage points (CI: 95% 78.4 ÷ 79.1 p<0.0001).

These observations show that in most cases, the decision aid provided by the CI protocols could be deemed useful by a clinician. Considering the number of findings or diagnoses made by the participants, the first five represent in most cases only one fifth of the answers (intended as findings) reported by the ECG readers, but they would have allowed for the recovery of 50% of the correct answers otherwise missed. Nonetheless, and despite the advantages of this second type of use of CI technologies, we note that the use CI methods (even when used as merely decision support tools) could have important consequences that are common to any other type of decision-making technology, including the risk of de-responsabilization, automation bias, or the deskilling of the involved clinicians Bond et al. (2018); Cabitza et al. (2017). Providing clinicians with a set of possible answers (similarly to what we did when we considered LA rather than SA), rather than a single most popular one, could limit the emergence of these unintended consequences by stimulating the clinician’s evaluation process. Nevertheless, further studies should aim to address which CI protocol could be more effective in avoiding, or at least limiting, these problems.

**Figure 5:**
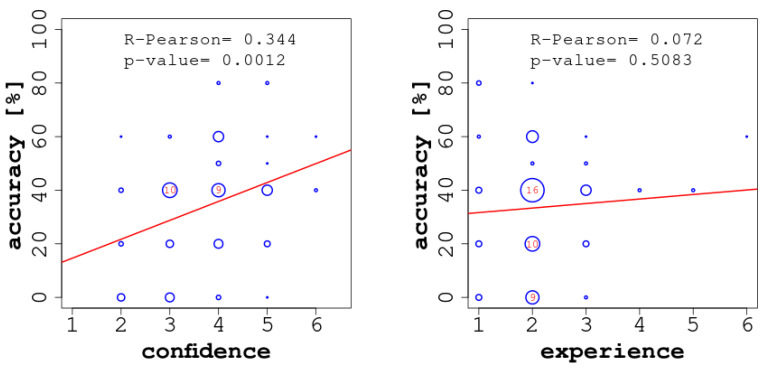
Correlations between accuracy and confidence (on the **left**) and between accuracy and experience (on the **right**). In each of the two diagrams, we report the Pearson correlation coefficient (ρ) and the corresponding *p*-value.

Considering the results in more detail, we observed a moderate-to-strong and statistically significant correlation between the accuracy of the individual readers and their confidence (see Figure 5, left). In our study, confidence acted as a good predictor of the readers’ accuracy, and this could provide a plausible explanation as to how the confidence-based aggregation protocol achieved the best results: the confidence of the individuals was often proportional to their accuracy and thus represented the best corrective. Further, and obviously, we did not observe any overlap between confidence and self-consistency in our study, which has been described and studied by Koriat and Bahrami Bahrami et al. (2010); Koriat (2012), due to the fact that the implemented CI protocols involved no interaction among the readers.

By contrast, we did not find a significant positive correlation between accuracy and experience (see Figure 5, right). Even though in the more complex ECGs (once seen—ever known, e.g., ECG 7) or in those ECGs most similar to everyday ECGs in terms of the quantity of findings (e.g., ECGs 5 and 6), more experienced readers were better able to focus on the most relevant findings; in more didactic exercises, and, contrary to our expectations, experience seemed to have a detrimental effect on performance (e.g., ECG 4). A possible explanation for this observation could be that the more ‘textbook-like’ cases were deemed ‘obvious’ by the students, while more experienced readers may have had a more nuanced perspective, which would entail multiple possible explanations. In fact, readers judged their ability to read the ECG on the basis of their daily experience.

It should not be surprising, therefore, that those who assessed themselves as more experienced performed better on those ECGs more similar to those encountered in everyday clinical practice. In this sense, our findings, while departing from what is reported in Kammer et al. (2017), do not necessarily contradict the idea that greater levels of experience imply greater diagnostic performance in clinical practice.

Finally, we note that in our experiments and in the reported results, we did not distinguish between expert (cardiology residents) and non-expert readers. This is because our experiment was primarily aimed at highlighting the potential of CI as a tool to reduce diagnostic errors through crowd-sourced second opinions. Although we deem it realistic that expert readers could also lend their opinions in support of such a service, we were curious to investigate what would change if, as is more usual in the CI literature, we removed the expert readers from the crowd and compared the performance of the resulting CI groups with that of the expert readers.

**Figure 6:**
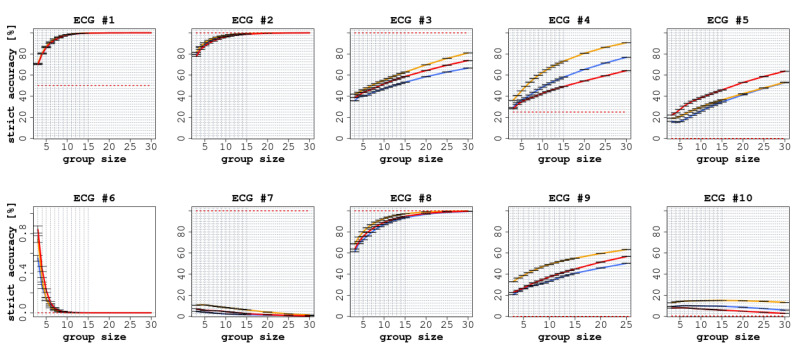
Strict accuracy (SA) on the 10 ECGs, with increasing group size, when comparing the expert readers (cardiology residents) against the groups of non-expert readers. In each graph, the red line represents the experience aggregation protocol, the yellow line represents the confidence aggregation protocol, and the blue line represents the frequency aggregation protocol. The red-dotted line represents the average performance of the individual expert readers.

**Figure 7:**
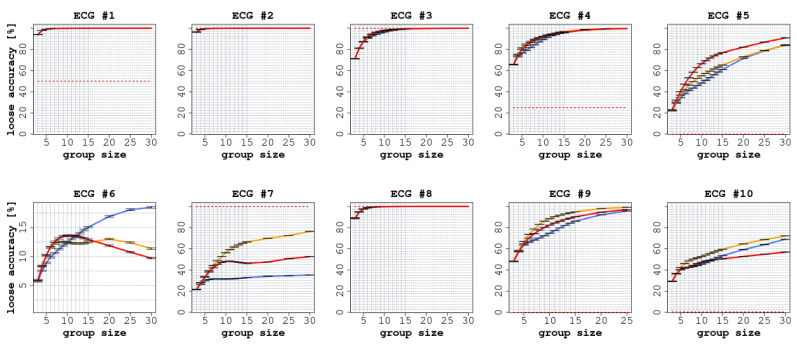
Loose accuracy (LA) on the 10 ECGs, with increasing group size, when comparing the expert readers (cardiology residents) against the groups of non-expert readers. In each graph, the red line represents the experience aggregation protocol, the yellow line represents the confidence aggregation protocol, and the blue line represents the frequency aggregation protocol. The red-dotted line represents the average performance of the individual expert readers.

The results of this experiment are reported in Figure 6 and Figure 7. ECGs 2, 3, 7, and 8 were correctly classified by the expert readers (i.e., both SA and LA were equal to 100%). Consequently, we observed a reduction in diagnostic performance for the groups of non-experts. The biggest such reduction was obtained on ECG 7 for both SA and LA (in the case of SA, specifically, the performance of the non-expert CI groups converged toward 0% as the group size was increased). By contrast, on ECGs 1, 4, 5, 6, 9, and 10, the non-expert CI groups were able to outperform the individual expert readers. Thus, in two thirds of the cases, the usage of CI allowed the groups of non-experts to rival or even surpass the accuracy of the individual experts.

**Figure 8:**
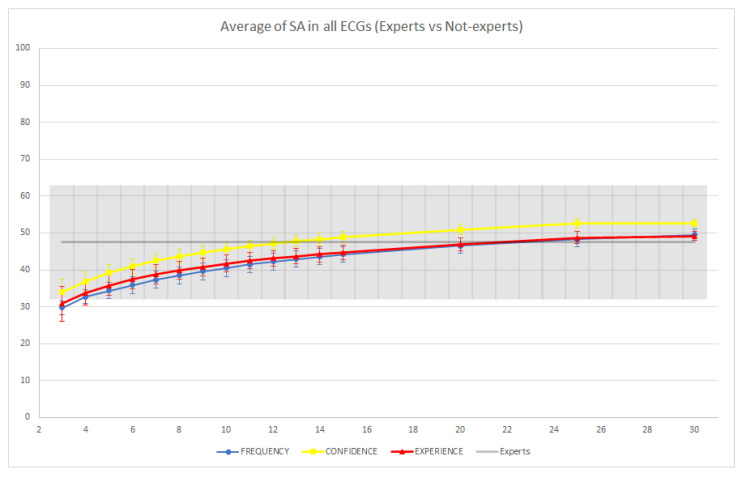
Average strict accuracy (SA) of individual expert readers and groups of non-experts across the 10 ECGs. The colored intervals and the gray-colored band represent the 95% confidence intervals.

**Figure 9:**
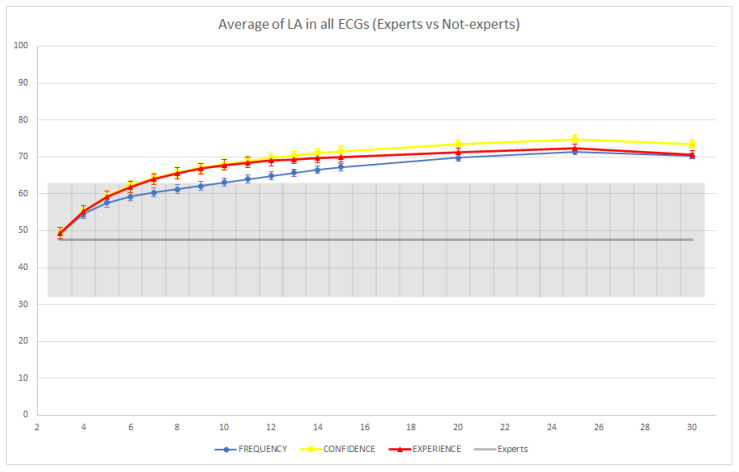
Average loose accuracy (LA) of individual expert readers and groups of non-experts across the 10 ECGs. The colored intervals and the gray-colored band represent the 95% confidence intervals.

In Figure 8 and Figure 9, we report the average performance of both the groups of non-experts and the individual expert readers across the 10 ECGs. In terms of SA, we observed that the average performances of the groups and individual experts were not significantly different (i.e., the confidence intervals overlapped). However, we observed a slight improvement of the confidence-based aggregation protocol in comparison with the performance of the experts when groups with more than 14 readers are involved, while the other two CI protocols seemed to require larger groups to achieve an improvement. In terms of LA, we observed that groups of non-experts involving as few as eight readers were able to significantly improve over the performance of the individual expert readers (using the confidence or experience-based CI protocols), and, on average, all CI groups were able to improve on the performance of individual readers. In conclusion, this shows that even without expert readers, the application of CI techniques could be useful to reduce diagnostic errors, and this reduction (with appropriately sized CI groups and CI aggregation protocols) could be even greater than what could be achieved through standard second reading protocols, which usually involve only a single (expert) reader.

### Limitations

This study has some limitations, as we partly anticipated above. First, there are issues with the representativeness of the sample regarding both the readers involved and the ECGs chosen. While the effects that we observed were statistically significant with respect to the size of the participant sample, no sample of ECGs, no matter how wide, could be fully representative of the whole body of examinations that a clinician could read in real settings. Therefore, the results cannot be generalized, notwithstanding their scientific relevance. However, we designed the study protocol to focus on some findings of potential interest for research on CI and the potential of this approach in ECG reading. We decided to involve students and young doctors not only because doing so is easier than enrolling established and busy specialists, but also because if platforms enabling CI exercises are to be established (also in light of studies similar to the present one), one could conjecture that these systems would enrol motivated students, residents, and young specialists who are willing to combine a learning-by-doing experience with a paid assignment. Alternatively, the involvement in such CI platforms could be proposed as a form of required practice exercise during medical studies. In this respect, we believe that such willingness to participate (which we observed in our experiment) and its influence on sample bias should be further investigated in the real-world applications of this protocol.

Second, we cannot rule out the impact of a *laboratory effect* Gur et al. (2008), according to which the raters in a simulated setting are expected to behave differently from how they would in a real-world situation, and likely worse, because they do not feel the need to achieve the demanding performance level that is required in a clinical environment when real lives are at stake. However, this effect can also be seen as a strength of the study, in that this would provide conservative insights on the positive potential of CI protocols in decision-making processes: In other words, whenever the participants of a similar initiative knew that their interpretation could induce real-impact effects, their performance would likely improve further and provide even better insights at the aggregate level. Further research should be aimed at the implementation and experimentation of such CI protocols in real-world medical practice, in order to very such a conjecture.

Third, there is also a potential bias of misclassification related by the manual evaluation of the responses to make them structured and further processable. However, to this respect, the availability of a reference gold standard (the WM database), enriched with adequate and didactic-oriented explanations and well-defined reading guidelines, should have mitigated this kind of bias.

Fourth, the number of answers that we considered in the computation of the LA construct was heuristically set to five in our study, as we deemed this amount sufficient to accurately describe all the cases that we considered. However, this threshold is arbitrary, and we are aware that a different one could have yielded slightly different results. In real-world settings, context-aware considerations should be made to choose a more apt number of responses, for instance, according to the ECG kinds, the case difficulty, the referring specialty and the expertise of the clinicians involved. Alternatively, rather than setting a pre-specified number of responses, the protocol could be defined by setting a bound on the probability of error (i.e., the probability that the correct answer, or the correct ones, is included in the collection of responses returned by the CI). In this case, the clinicians interrogating the crowd would simply state that they prefer to receive a set of alternatives that are associated with a probability of at least, say, 95%, and statistical inference or AI-based techniques, such as conformal prediction Balasubramanian et al. (2014) or three-way decision-making Campagner et al. (2020), could be used to comply with this requirement. However, we did not employ these advanced techniques in our study, leaving it to future work.

Lastly, while in this article we focused on CI, we should note (as briefly hinted at in Section 1) that other decision support technologies, with the same goal of reducing diagnostic errors, have also been proposed, chiefly among them being the so-called *artificial intelligence* (AI). Indeed, promising results have shown that machine learning (ML)-based AI systems can achieve performance levels similar to (or better than) human clinicians Liu et al. (2019), and such research has largely increased the popularity of AI methods in clinical settings, including ECG reading Hong et al. (2020); Murat et al. (2020). Here, we briefly mention some differences between the two approaches and the implications of our results. The first difference regards the workloads required by the two approaches. While AI can be much more efficient than CI when evaluating a new case, the development of AI systems (especially those based on deep learning) usually requires large annotated training sets and high-performance hardware, and it may have significant computational and environmental costs Strubell et al. (2020). The second difference regards interpretability. Indeed, despite their accuracy, the inner workings of AI methods are not easily interpretable by humans (i.e., the so-called black-box problem). This is a limitation that could have important ethical and legal consequences Vayena et al. (2018); Watson et al. (2019) and one that may severely hinder the applicability of such systems in the medical setting. In contrast, CI techniques can achieve increased interpretability compared to AI systems; even though traditional CI methods have so far mainly relied on *closed-ended answers*, which may not be interpretable, the approach that we discussed in this paper (which is based on free-ranging text annotations), or techniques based on interactive CI protocols, may achieve a level of interpretability comparable to standard second-reading protocols. Further work should therefore be aimed at exploring this line of research. Notwithstanding these differences, both approaches have been shown to be effective at reducing diagnostic accuracy. In fact, recent studies Cabitza et al. (2021); Patel et al. (2019) have shown that hybrid techniques (combining group decision-making and AI) could be even more effective than state-of-the-art AI and CI approaches while retaining the advantages of both methods. For this reason, we think that future research should further explore these hybrid decision support strategies Peeters et al. (2020).

Moreover, AI and CI can also converge in the case of using CI protocols for *ground truth vetting* Liang et al. (2017). In this setting, we have a ground truth that has been established by one or more experts, and the CI is used so as to detect, validate, and possibly correct potential errors in the ground truth labeling. Cases for which there would be a large disagreement among the readers in the crowd, when there is a strong disagreement between the majority options and the ground truth labels, or also when the crowd strongly supports an answer which is different from the one in the available ground truth could be reported by the CI platform as possibly affected by label bias Wiens et al. (2020). Accordingly, caution would be recommended or a careful re-examination of the suspect cases would be promoted. This in turn would enable the establishment of sounder ground truths, which would be useful not only for daily practice or education, but also for the development of ML systems, which are strongly impacted by data quality issues Cabitza et al. (2019); Campagner et al. (2020).

## 5. Conclusions

In this article, we studied the application of CI to the task of ECG reading and discussed the more general implications of this approach in medicine for diagnostic practices and more general decision-making tasks. CI protocols allow for the opinions of a multitude of expert individuals to be combined together without direct interaction; data can be collected from this collective in a wide range of ways, including personal tools that are connected to the internet, such as smartphones and computers. The results presented in this study corroborate the idea that CI protocols, empowered by current IT, can support healthcare practitioners in making better interpretations and decisions by leveraging the best ‘second opinion’ (or set of plausible second opinions) that are made available by aggregating, with different techniques, the interpretations of multiple readers. Among these techniques, we detected a clear potential for the protocols that exploit additional information about the experiences of the raters involved and, most notably, their confidence in their indications.

As reported in Section 3, the application of CI protocols led to a significant improvement in terms of the diagnostic error rate compared to individual readers and small groups, even when we considered groups composed only of non-experts. In particular, we found that the most effective CI protocol was the confidence-weighted majority. This suggests that successful CI initiatives need protocols that are aptly tuned to leverage more information than just single decisions, like self-reported expertise and perceived confidence in the single decision.

In light of these results, as well as the wide use of ECGs in clinical practice, we claim that CI can be considered an effective solution to reduce the occurrence of diagnostic errors in ECG reading. Due to the essentially asynchronous and distributed nature of CI approaches, these latter ones could be applied even in small centres where specialist readings or second opinions by specialists can be difficult to obtain. This is especially true also in primary settings (i.e., for non-urgent and screening cases), where the aggregate response ‘from the crowd’ can be combined together within reasonable time limits. In all these cases, we envision CI solutions as a convenient and relatively inexpensive means to augment the intelligence (i.e., interpretative accuracy) of a single clinician, who refers a difficult case to a small *extemporaneous-asynchronous-and-distributed* team of medicine students, novices or residents and gain a sort of superhuman ‘second opinion’ from such a ‘crowd’ Scalvini et al. (2011).

In light of the human and economic costs related to diagnostic and therapeutic errors, further research should be pursued in this area to assess and detect the best ways to actually integrate the potential of CI protocols into daily clinical practice. In particular, the costs and benefits of such CI systems should be further assessed; while we conjecture that appropriate incentives, or encouraging medical students to use these platforms as an alternative or complementary form of training, could allow for large numbers of redundant readings to be obtained without an excessive increase in terms of costs, further research must be aimed at determining the exact nature of these trade-offs in economical, technological, and computational terms. Further studies are also necessary to determine which CI protocols and rules are most effective (thus verifying our promising findings about the confidence-weighted protocols), which online platforms are more feasible and cost-effective to engage multiple raters effortlessly, and which incentives for the participants would make these initiatives sustainable in the long run, so that the results achieved and observed in experimental settings, such as ours, could be replicated in real-world contexts.

## Figures and Tables

**Table 1:** Description of the 10 ECGs employed in the study. Legend: STEMI = ST-segment elevation myocardial infarction; PE = pulmonary embolism; AF = atrial fibrillation; RVH = right ventricular hypertrophy; EAR = ectopic atrial rhythm; AV = atrioventricular; PSVT = paroxysmal supraventricular tachycardia.

ECG ID	MW Reference	MW Difficulty	Evaluated Difficulty	Pathology Class
1	251	1/5	Easy	Normal
2	91	1/5	Easy	STEMI
3	3	3/5	Medium	Pericarditis
4	32	3/5	Medium	PE
5	18	4/5	Difficult	AF + RVH
6	6	4/5	Difficult	EAR + AV Block II Mobitz 1
7	285	3/5	Medium	Hypothermia
8	323	2/5	Medium	PSVT
9	108	5/5	Medium	STEMI
10	19	4/5	Medium	Dextrocardia

**Table 2:** Number of readers and distribution of their expertise level for each ECG.

ECG ID	Readers	Senior Students	Recnt Graduates	Residents (Not Cardiology)	Residents (Cardiology)
1	117	32.5%	48.7%	17.1%	1.7%
2	140	22.1%	50.7%	22.1%	4.3%
3	91	31.9%	49.5%	16.5%	2.2%
4	88	20.5%	55.7%	19.3%	3.4%
5	88	31.8%	47.7%	18.2%	2.3%
6	97	19.6%	54.6%	20.6%	4.1%
7	80	32.5%	46.3%	18.8%	2.5%
8	102	20.6%	53.9%	19.6%	4.9%
9	75	30.7%	49.3%	17.3%	2.7%
10	86	19.8%	55.8%	20.9%	2.3%

## Data Availability

Experimental data is available from the authors upon reasonable request.
